# Right upper lobe pulmonary sequestration masquerading clinically and radiologically as malignancy: a case report

**DOI:** 10.1093/jscr/rjad022

**Published:** 2023-01-31

**Authors:** Ankit Gupta, Gavin Chetty, David Hopkinson, Jagan Rao, Govind Chetty

**Affiliations:** School of Medicine, Worsley Building, University of Leeds, Woodhouse, Leeds, LS2 9JT, UK; Northern General Hospital, Herries Road, Sheffield S5 7AU, UK; Northern General Hospital, Herries Road, Sheffield S5 7AU, UK; Northern General Hospital, Herries Road, Sheffield S5 7AU, UK; Northern General Hospital, Herries Road, Sheffield S5 7AU, UK

## Abstract

Bronchopulmonary sequestration is a rare disease in which a non-functional region of pulmonary tissue receives an aberrant vascular supply and lacks normal communication with the tracheobronchial tree. We present the case of a 30-year-old female with a primary complaint of unexplained weight loss and no other additional signs or symptoms. In view of this, computed tomography imaging was ordered, showing a 33HU mass in the right upper lobe. A specialist radiologist reviewed the images and concluded that the most likely differentials were mediastinal lymphoma or thymic malignancy. Video-assisted thoracoscopic surgery was performed, when it was seen that no malignancy was present, but rather a bronchopulmonary sequestration. Histology confirmed the diagnosis; the patient fared well post-operatively. Bronchopulmonary sequestration is a rare pathology, with most cases occurring in the lower lung lobes. This case is highly atypical, due to the lack of clinical features and the lesion radiologically mimicking the appearance of malignancy.

## INTRODUCTION

Discovered by Rokitanski and Rektorzik in 1861, bronchopulmonary sequestration (BPS) is an isolated mass of non-functioning lung tissue, complete with alveoli and bronchi. The bronchial tree does not communicate with the mass, and sequestrations are typically classified as: extralobar, when encased by its own pleural investment, or intralobar, if lacking a membrane. BPS often occurs in the lower left lobe but may occur in posterior lung segments and in or below the diaphragm. Arterial supply is aberrant, usually arising as branches from the aorta, subclavian, intercostal or diaphragmatic arteries [1].

We report a rare case of right upper lobe (RUL) BPS mimicking a malignancy, and its successful removal by video-assisted thoracoscopic surgery (VATS).

## CASE REPORT

A 30-year-old female presented to her general practitioner with a chief complaint of unexplained weight loss over several months. The patient was otherwise well, denying any other symptoms, including B symptoms. Physical examination was unremarkable.

**Figure 1:**
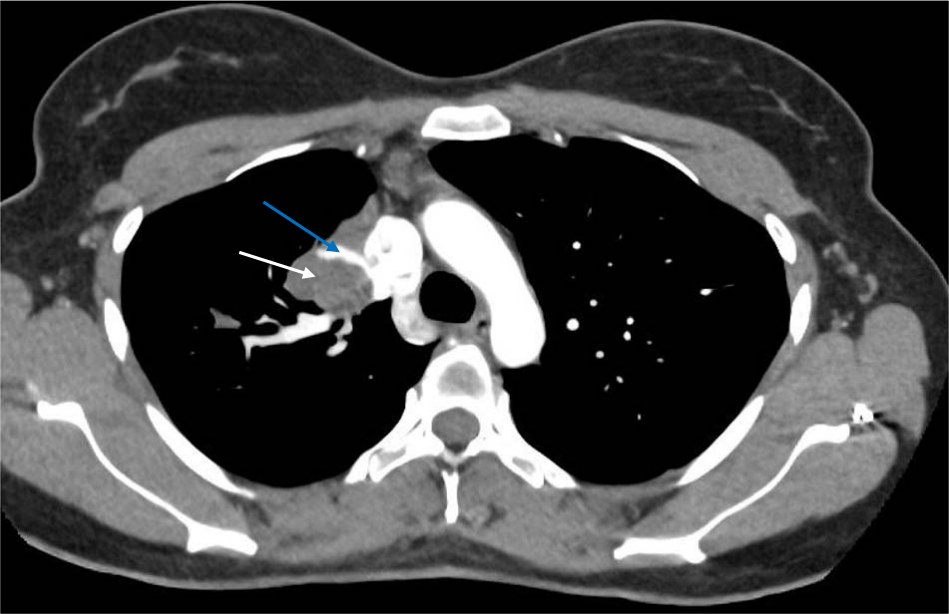
Axial arterial phase CT scan demonstrating pulmonary sequestration (bottom arrow). A large venous branch draining into the azygous vein can be identified (top arrow).

**Figure 2:**
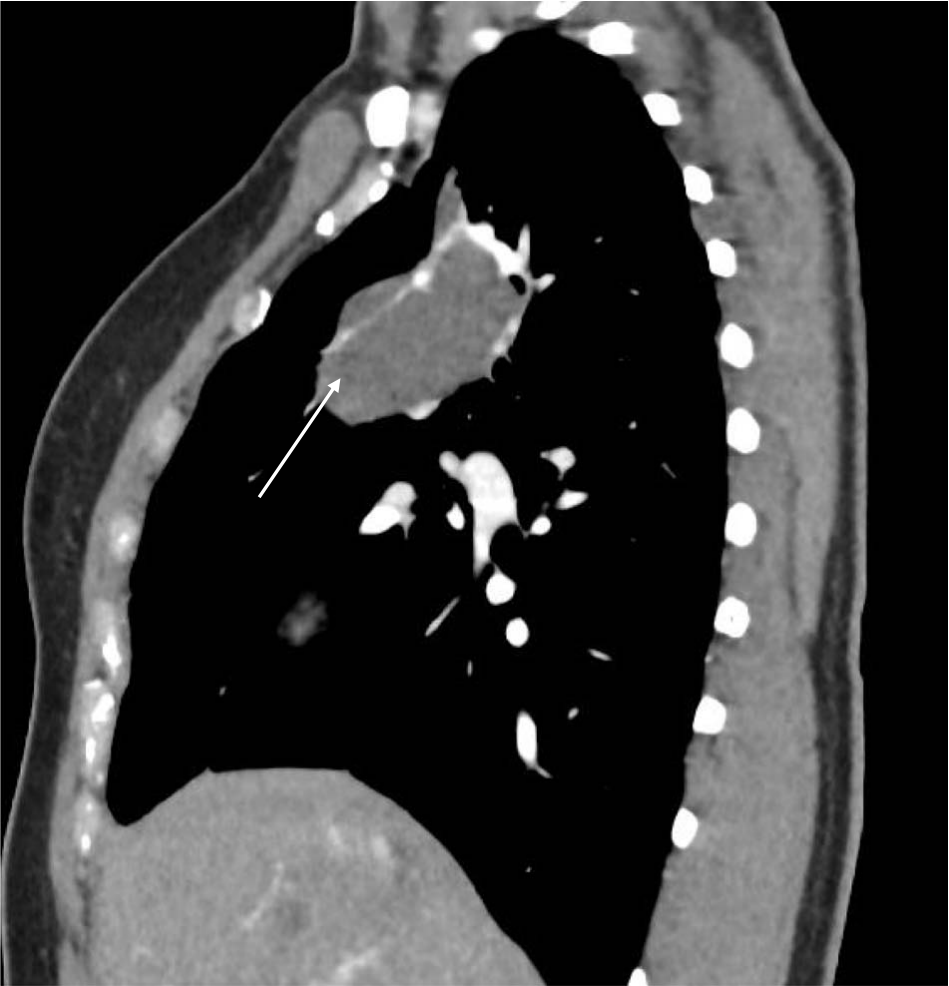
Sagittal CT scan demonstrating pulmonary sequestration in the right upper lung lobe (arrow).

Computed tomography (CT) scans demonstrated a well-demarcated right-sided 5.5 × 2.6 cm paramediastinal solid mass, with associated atelectasis of the RUL (Figs 1 and 2). Traversing this segment, a large venous tributary of the azygous vein was identified (Fig. 2).

The top differential diagnoses suggested at this stage were mediastinal lymphoma or thymic malignancy. The mass had an average attenuation of 33HU, in keeping with such differentials. It was noted that the thymus was separate to the lesion. The specialist radiologist from the referring centre was confident that this was a solid mass, based upon the radiological findings.

**Figure 3:**
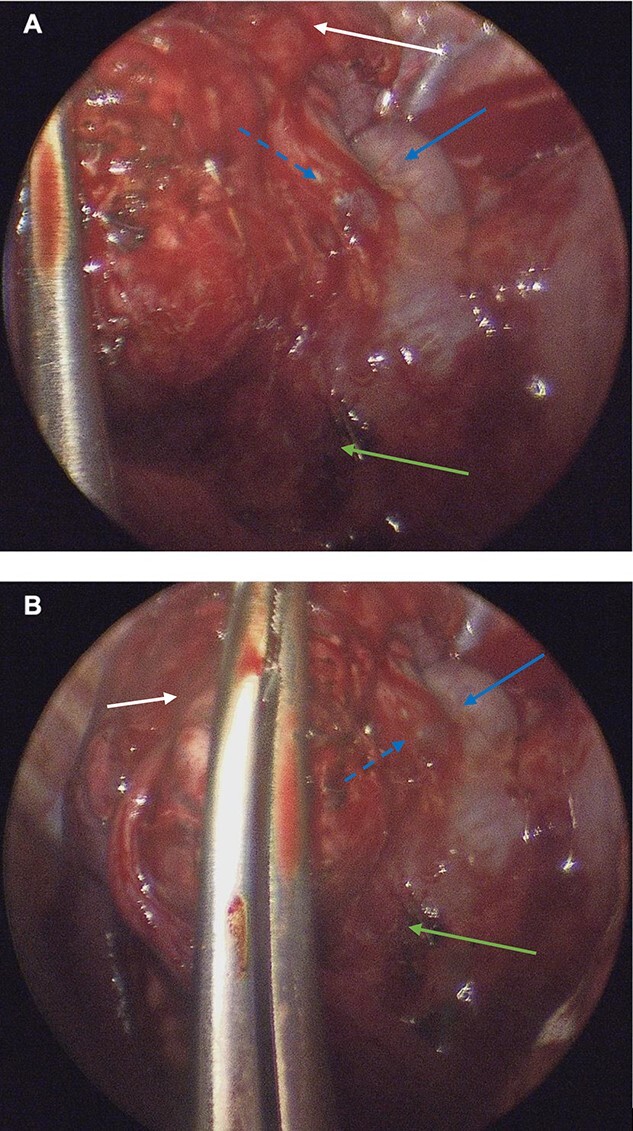
(**A**, **B**): Intraoperative thoracoscopic images demonstrating pulmonary sequestration (top arrow). A venous branch (middle punctuated arrow) of the azygous vein (middle solid arrow) may be appreciated. A surgical clip was placed on a systemic feeding artery (lowest arrow).

A single-port access in the fifth intercostal space along the mid-axillary line was used for VATS. Multiple adhesions from the RUL were released with diathermy, at which point it became evident that no paramediastinal mass was present, but rather, the pathology was located within the lung parenchyma itself. The azygous vein was distended, and the texture of the RUL lung tissue differed from the other lobes. The impression of the surgical team was that this was a rare case of RUL BPS (Fig. 3).

Upon mobilizing the lesion from the mediastinum, the systemic arterial feeding vessels were ligated and divided. The proximal extent of the lesion lied close to the segmental pulmonary artery branches, and there was concern that the staple would fail if the vessels were stapled en masse. Thus, these branches were divided individually. The staple line encompassed segmental lung parenchyma and some sub-segmental bronchi. In essence, the operation was not a classical anatomical segmentectomy, but was necessary to resect the BPS in its entirety. After resection, the lower, middle and remaining segments of the upper lobe inflated well with no atelectasis. A single chest drain to the apex was inserted, and the VATS port site was closed in layers with Vicryl.

**Figure 4:**
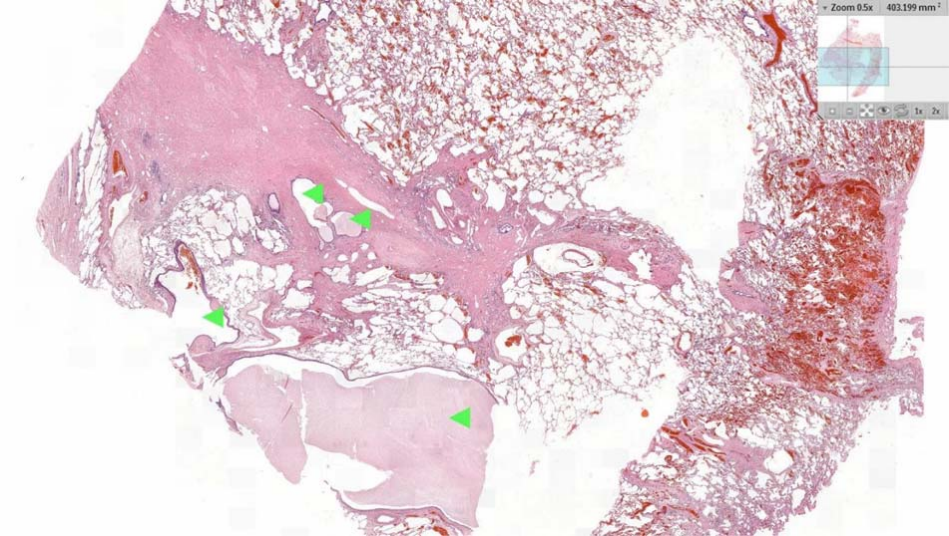
Histology image of the resected pulmonary section. Dilated bronchial spaces with mucous impaction can be identified (arrows).

Histology of the specimen was undertaken to confirm the diagnosis. On sectioning, the lung parenchyma was extensively pneumonitic, and areas of fibrosis were identified surrounding blind and dilated bronchial spaces (Fig. 4). Haemorrhagic congestion with thickening of the vasculature was distributed within the fibrosed areas. Full histological analysis demonstrated changes consistent with BPS. Ultimately, no evidence of dysplasia or malignancy were found.

**Figure 5:**
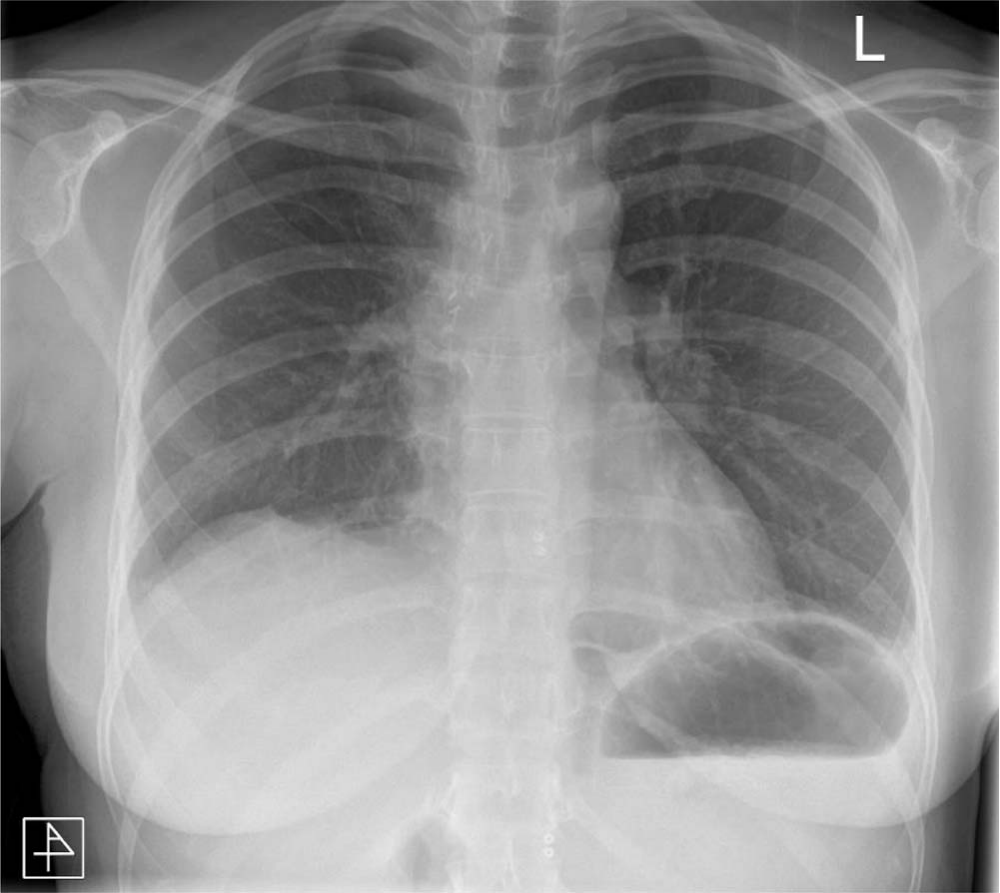
Anteroposterior post-operative chest radiograph.

The post-operative course was unremarkable. The chest drain was removed on Day 2, and the patient was discharged on Day 3. The patient has since been reviewed in clinic and is well. Follow-up radiographs demonstrated absence of any remaining pathology (Fig. 5).

## DISCUSSION

BPS is a rare anomaly, characterized by non-functional pulmonary tissue receiving an aberrant vascular supply and lacking communication with the tracheobronchial tree. Savic *et al*. found that 97.75% of 400 BPS cases occurred in the lower lobes, with only eight cases of upper lobe pathology [2]. Here, we present a rare case of RUL BPS, masquerading as malignancy, both clinically and radiologically.

Embryologically, such lesions are classified as foregut malformations and diagnosed via prenatal ultrasound or CT. Although rare, communicating bronchopulmonary foregut malformations can lead to sequestration, accounting for <6% of congenital lung malformations [3]. It is theorized the formation of sequestrations is due to an accessory lung bud that continued to migrate with the oesophagus caudally, acquiring a blood supply from the primitive splanchnic vessels. Commonly, intralobar sequestrations present as recurrent segmental pneumonia. Other clinical features include chest pain, dyspnoea and haemoptysis [4].

Although benign, a prompt diagnosis is important in facilitating timely treatment to prevent malignant changes, abscess formation, bronchiectasis, torsion and other serious sequelae. Nonetheless, diagnosis is difficult, and most cases are diagnosed post-operatively via histology [5].

Literature states that misdiagnosis of BPS is common, with an average incorrect pre-operative diagnosis rate of 58.63% [4]. Previously, digital subtraction angiography (DSA) was considered the gold-standard diagnostic test, as it explicitly shows anomalous arterial supplies [6]. However, DSA is invasive and involves significant radiation exposure. Contrast CT is now regarded as the radiological test of choice, as non-contrast CT does not consistently show anomalous arterial supplies [7].

Treatment of BPS is often curative via surgical resection, especially in those who are symptomatic. Some authors argue that a ‘wait-and-watch’ approach may be used for asymptomatic patients; however, this is an area of contention [8, 9]. Certainly, there are reports of patients developing complications of BPS such as infection and abscess formation; yet, perhaps most pertinently, there are numerous documented cases of near-fatal haemoptysis and one case ending in fatal haemoptysis [10–12].

The atypicality of our case conveys an important message to clinicians. A lack of clinical clues makes even the suspicion of BPS difficult, let alone a diagnosis. No specific arterial branch was seen to supply the lesion, except for a few branches from the pulmonary artery. Perhaps when diagnostic uncertainty exists with a lung mass, further investigations such as magnetic resonance angiography or DSA should be performed to accurately ascertain a diagnosis, allowing for tailored management.

To conclude, BPS is a rare thoracic abnormality that may mimic sinister malignancies, both in terms of clinical and radiological features. A high index of suspicion is pivotal in making the diagnosis. Clinicians must consider BPS as a differential diagnosis if patients lack typical cancer symptoms and have a lung lesion with aberrant vascular connections upon imaging.

## CONFLICT OF INTEREST STATEMENT

None declared.

## FUNDING

None.
